# Automated screening of research studies for systematic reviews using study characteristics

**DOI:** 10.1186/s13643-018-0724-7

**Published:** 2018-04-25

**Authors:** Guy Tsafnat, Paul Glasziou, George Karystianis, Enrico Coiera

**Affiliations:** 1 Centre for Health Informatics, Australian Institute of Health Innovation, Macquarie University, Sydney, Australia; 2 Centre for Research in Evidence-Based Practice, Bond University, Gold Coast, Australia; 3 Kirby Institute, University of New South Wales, Sydney, Australia

**Keywords:** Automation of systematic reviews, Evidence screening, Study selection, Study characterisation

## Abstract

**Background:**

Screening candidate studies for inclusion in a systematic review is time-consuming when conducted manually. Automation tools could reduce the human effort devoted to screening. Existing methods use supervised machine learning which train classifiers to identify relevant words in the abstracts of candidate articles that have previously been labelled by a human reviewer for inclusion or exclusion. Such classifiers typically reduce the number of abstracts requiring manual screening by about 50%.

**Methods:**

We extracted four key characteristics of observational studies (population, exposure, confounders and outcomes) from the text of titles and abstracts for all articles retrieved using search strategies from systematic reviews. Our screening method excluded studies if they did not meet a predefined set of characteristics. The method was evaluated using three systematic reviews. Screening results were compared to the actual inclusion list of the reviews.

**Results:**

The best screening threshold rule identified studies that mentioned both exposure (E) and outcome (O) in the study abstract. This screening rule excluded 93.7% of retrieved studies with a recall of 98%.

**Conclusions:**

Filtering studies for inclusion in a systematic review based on the detection of key study characteristics in abstracts significantly outperformed standard approaches to automated screening and appears worthy of further development and evaluation.

## Background

The relentless growth in research and the greater reliance placed on evidence for clinical and policy decisions have created a need for automation to support the process of systematic reviews [1, 2]. A core process in the creation of such reviews is the initial search for and selection of research articles for inclusion in the review. Often a literature search identifies hundreds to thousands of candidate documents, with the vast majority being screened and then excluded because they are not relevant to the review question.

To save time and resources on screening, often only the title and abstract are appraised first, and the more taxing task of appraising the full text is reserved for cases where decisions cannot be made based on the abstract alone, where reviewers disagree on whether to include or exclude, and for those papers that appear relevant at title/abstract screening, but may be disregarded at full-text screening [3]. However, screening on the abstract and title is likely to be the most time-consuming of all systematic review tasks because of the large number of references typically retrieved from the high-sensitivity search strategies employed in systematic reviews [4].

Several supervised machine learning algorithms have been tested to automate screening. A recent review found 44 such algorithms [5], and this functionality is already available in several commercial systems. These algorithms use natural language processing to determine the probability that a candidate paper should be excluded from a systematic review. Machine learning systems work by training algorithms based upon the inclusion and exclusion decisions made by human reviewers and then creating a classifier that models these observations. A limitation of such machine learning screening systems is the large number of human decisions that are needed before a reliable classifier can be developed. A second limitation is that the reliability of classifications appears relatively low. These algorithms are thus used to exclude only the most obvious cases (between 30 and 70% of candidate studies) and often only rank studies in a decreasing order of likelihood in preparation for human screening. For example, the systematic review tool Rayyan updates a machine learning classifier that promotes abstracts to the top of the screening queue when they have more similar words to previously included abstracts [6].

Whilst it makes sense to model automation on human processes [7], there is no requirement that each step of automation in screening must be identical in their human and machine versions. What is time-consuming for humans may be easy for a machine. For example, the extraction of key information from an abstract about the characteristics of a study (such as the population, exposure, confounders and outcomes) only occurs after screening in the human process, because such extraction is time expensive for humans.

No such time costs however hold on the machine version of screening. Our hypothesis for this study is that automated extraction of study characteristics from abstracts can itself be used to make screening decisions. This effectively re-orders the tasks in a human-driven systematic review so that extraction of key information (step 3, see Table 1) occurs before screening (step 2). In this study, we test to see if automated information extraction can lead to effective screening and thus reduce the overall screening load on humans.

**Table 1:** High-level steps of a systematic review

Step 1: Conduct a broad search of the literature	
Step 2: Screen the search results for relevant articles and exclude all others	
Step 3: Extract study characteristics from included studies	
Step 4: Synthesise the studies based on extracted characteristics and report on findings	

Information extraction techniques that combine supervised machine learning and heuristic methods appear to be a promising approach to this task [8]. This has been aided by the increased use of clinical trial registries and specialised databases (e.g. Epistemonikos [9]) which means that more easily managed structured information is available for screening algorithms.

## Materials

### Data preparation

Due to limited currently available data, and limited resources in generating such data, we have made secondary use of data already available to us from a previous study conducted in our group [10]. The fact that the same data was used to develop and evaluate the semi-automated characteristic extraction method used here did not bias our results as the extraction algorithm is only meant to *simulate* an automated extraction method that is not part of this study. We thus identified three recent systematic reviews of environmental observational studies (Table 2) that included a defined and repeatable search strategy.

**Table 2:** The three systematic reviews used in this study

Name	*O*	*N*	*n*	*I*	Topic
Hamra 2014 [14]	604	615	615	17 (2.7%)	Outdoor particulate matter exposure and lung cancer
Johnson 2014 [15]	3023	3023	2470	17 (0.7%)	PFOA effects on fetal growth
Thayer 2013* [16]		2054	1880	11 (0.6%)	Bisphenol A (BPA) exposure and obesity

The list of relevant studies was provided by the authors

*O* original number of articles reported in the original review, *N* number of articles in our search results, *n* number of articles with abstracts, *I* number of included articles in the systematic review

*Thayer 2013 provides a search protocol and no search results

For each review, we consulted with the corresponding authors to ask for the original search results they used, to ensure that the search strategy provided in the review is indeed the correct one and asked for their search results. When the original search results were not available from the authors, we have repeated the search strategies using the databases specified in the original searches, using the limit dates of the original search. The original search queries and a comparison between the original searches and the reproduced searches are given in Appendix 2.

The citations for articles identified in the searches were then collected using EndNote (EndNote X7.7.1; Bld 10036). Duplicate entries were removed using EndNote’s de-duplication function. Abstracts were retrieved automatically using EndNote’s “Find Reference Updates…” function and references without abstracts were removed. Five of the included studies in Thayer were removed as a result of this process. References with abstracts were then exported to text files in preparation for automated extraction.

### Study characteristic extraction

We used a previously developed text mining algorithm to extract six study characteristics of observational studies: population, exposure, confounders, outcomes, (collectively PECO), country and study type. The algorithm was developed using the General Architecture for Text Engineering (GATE) [11]. The algorithm’s accuracy has been tested previously on the included studies in these systematic reviews with precision = 95% and recall = 81% (*F* score = 87%) [10].

The algorithm uses human-crafted grammatical rules designed to suit the abstracts of observational studies. Study characteristic recognition is performed by first identifying semantic elements using specific dictionaries and semantic rules. The rules and dictionaries were developed manually by inspecting the 17 articles included in Johnson 2014 (training set). The rules were further tested using 34 articles that include the 17 articles included in Hamra 2014 and tested using 35 articles including the 11 of the 16 included in Thayer 2013. In each case, the exposure and outcome dictionaries were updated for the corresponding review question. The identification algorithm dictionaries and rules are described in Appendix 1.

The output from the extraction algorithm is text phrases that match the attributes of the six study characteristics. However, for the purpose of this study, we ignored this text output and only noted whether or not the algorithm had found candidate phrases for a given characteristic from an abstract. Whenever a required PECO item was identified, this was recorded as a “hit”, and if no phrases could be identified, this was coded as a “miss”.

### Screening evaluation

We tested several variants of screening rules which applied different thresholds for the number of study characteristics needed to include a study in a review (Table 3). These threshold rules ranged from a strict rule that required all four PECO items to be detectable, to rules that required some subset of elements to be detectable. We applied each of the six extraction rules to abstracts identified in the search strategy.

**Table 3:** Screening rule tests in this study and the rationale behind each

No.	Screening rule	Rationale
1	All 4 PECO Terms	A well-written abstract of an observational study should mention all PECO elements. PECO elements are chosen (and not country and study type) because these are regularly used to retrieve studies relevant to a systematic review question.
2	Any 3 PECO Terms	We expect a higher recall and lower precision than the rule 1 as this allows one PECO element to be missed.
3	Any 2 PECO Terms	We expect a higher recall and lower precision than the rule 2 as this allows an additional PECO element to be missed.
4	PEO	We expect the same or lower recall and higher precision than rule 2 because confounders are often omitted from observational study abstracts.
5	PE	We expect the same or lower recall and higher precision than rule 3 because of an assumption that abstracts of observational studies should mention the population the study was conducted on and the exposure that was measured.
6	EO	We expect the same or lower recall and higher precision than rule 3 because of a belief that abstracts of observational studies should mention the exposure that was studied and the outcomes that were observed.

Included articles that met all the requirements of a screening rule were counted as a true positive (TP). Those that met the screening rule requirements but were excluded in the original systematic review were considered false positive (FP). Articles included in the original systematic review that did not meet a screening rule criteria were counted as false negatives (FN). The remaining references that did not meet the screening rule and were excluded from the original systematic review were counted as true negatives. We calculated the precision (Pr) and recall (Re).

We assume that all articles not excluded by the screening algorithm (i.e. FP and TP) are to be manually screened. We thus estimated the “work saved” as 1 − (TP + FP)/*N* where *N* (or *n*) is the number of articles that were screened. This assumption also means that each FN article is an important study that will not be manually screened and would thus be excluded from the review. Hence, we consider perfect recall (i.e. Re = 1.0) to be of the highest importance and precision to be of secondary importance.

We calculated the maximum work saved as the proportion of manual screening that would need to be done after a theoretical perfect screening tool that has perfect recall and perfect precision. The maximum work that can be saved for each review is 1 − TP/*N*.

## Results

There was a wide range of result among the six rules but consistent results for each rule across the three evaluation sets. Table 4 shows the screening results for each of the rules on each of the reviews. The table also shows the maximum work that can be saved with perfect recall for each review. Overall, the best screening rule was EO as it provided the best recall with the highest precision and thus most work saved.

**Table 4:** Summary of screening workload savings for three systematic reviews

Screening rule	TP	FP	FN	TN	Pr	Re	Work saved
Hamra 2014 (*n* = 615 articles)							Max = 97.2%
All 4 PECO Terms	5	5	12	593	50%	29%	98.4%
Any 3 PECO Terms	12	24	5	574	33%	71%	94.1%
Any 2 PECO Terms	17	89	0	509	16%	100%	82.8%
PEO	11	17	6	581	39%	60%	95.4%
PE	11	13	6	585	46%	65%	96.1%
EO	17	65	0	533	21%	100%	86.7%
Johnson 2014 (*n* = 2470 articles)							Max = 99.3%
All 4 PECO Terms	3	1	14	2455	75%	18%	99.8%
Any 3 PECO Terms	14	12	3	2441	54%	82%	98.9%
Any 2 PECO Terms	16	60	1	2393	21%	94%	96.9%
PEO	13	49	4	2413	25%	76%	97.5%
PE	13	5	4	1551	72%	76%	99.3%
EO	16	11	1	2442	59%	94%	98.9%
Thayer 2013 (*n* = 1880 articles)							Max = 99.4%
All 4 PECO Terms	7	20	13	1840	26%	35%	98.6%
Any 3 PECO Terms	9	83	2	1786	10%	82%	95.1%
Any 2 PECO Terms	11	304	0	1565	3%	100%	83.2%
PEO	7	116	4	1753	6%	64%	93.5%
PE	14	45	6	1815	24%	70%	96.9%
EO	11	195	0	1674	5%	100%	89.0%
Average (*n* = 4965 articles)							Max = 99.1%
All 4 PECO Terms	15	26	39	4888	37%	28%	99.2%
Any 3 PECO Terms	35	119	10	4801	23%	78%	96.9%
Any 2 PECO Terms	44	453	1	4467	9%	98%	90.0%
PEO	31	182	14	4747	15%	69%	95.7%
PE	38	63	16	3951	38%	70%	98.0%
EO	44	271	1	4649	14%	98%	93.7%

Work saved is the proportion of all positives in the entire set of n references (i.e., 1 − (TP + FP)/*n*)

*TP* true positive, *FP* false positives, *FN* false negative, *TN* true negative, *P* precision, *Re* recall

The *All 4 PECO Terms* screening rules generally performed worst. Screening with *Any 3 PECO Terms* improves recall but only screening on *Any 2 PECO Terms* provides the desired recall. Comparing the screening results of the *All 4 PECO Terms* rule with those from the PEO rule, we can see that about half the misses of the former seem to be due to confounders not being mentioned in the abstract. The *PE* rule improved precision compared to the *Any 2 PECO Terms* rule, but had a worse recall, whereas the *EO* rule had the same recall as the *Any 2 PECO Terms* with a higher precision at every case.

Using the best screening rule (*EO*), the screening load reduction was between 86.7% (out of maximum possible of 97.2%) and 89% (out of maximum possible 99.3%) for Hamra 2014 and Thayer 2013, respectively, and over 98% (out of maximum possible 99.4%) for Johnson 2014 (Table 4). We note that Johnson 2014 also did not achieve perfect recall because one included study was missed (i.e. was classified as a false negative). The more generalised *Any 2 PECO Terms* screening rule also missed the same paper; hence, the overall FN rate across all three datasets was 2%.

## Discussion

Screening studies based on study characteristics is an effective way to address the need for automation of screening references as part of a systematic review.

A recent systematic review of automatic screening methods [5] found that all 44 reviewed methods used machine learning and were observed to save between 30 and 70% of screening decisions with up to a false positive rate (FPR) of 5% (i.e. Re = 95%). A more recent review of these methods questions the confidence in these results due to the similarity of the methods and the wide range of results [12].

By comparison, the use of automatically detected study characteristics in abstracts appears to be even more effective and reliable. For example, screening by detection of exposure and outcome (EO) mentions in an abstract in this study saved 93.7% of the screening work with 2% FPR (Re = 98%). In other words, in a systematic review with 10,000 articles to screen, automatically screening 70% of articles leaves the reviewer 3000 articles to screen manually. A system that screens 93.7% would leave the reviewer 630 articles to review manually, almost five times less.

Whilst this method appears to dramatically reduce reviewer screening effort, it does generate some additional work in algorithm development. Specifically, the information extraction algorithm used here is not fully automated, and some effort had to be put into developing two dictionaries specific to the topic of the reviews and may require even more effort when applied to other types of articles (e.g. RCTs). This work focuses on the potential of extraction algorithms to assist in screening and not the extraction algorithm itself.

The approach of using study characteristics for screening depends on having a reliable method for identifying study characteristics. A recent review of text extraction methods for study characteristics [8] showed that most algorithms focused on identifying the sentences that hold key information, rather than automatically extracting the information. We anticipate that new extraction algorithms will greatly improve the extraction of trial characteristics from abstracts.

An alternative approach to characteristic extraction using text mining is to take advantage of trial registries. Registries typically will report in a structured format the kind of study characteristics used in this study, and these could be automatically identified without the need for text mining and concept or word recognition, at least for screening using the approach used here. The number of clinical trials that can be found in online registries is increasing, and the quality of the data in these registries is improving. Other databases of clinical trials that contain trial characteristics (e.g. Epistemonikos [9]) are another potential source that can be used for screening although, in this study, we did not assess how these would be used.

### Error analysis

We have analysed the abstract of the one study that was missed by the system [13]. Of the PECO elements, the abstract actually mentioned the exposure and population (and country) but not the outcome or any confounders. However, both exposure and population were described in terms that were missing from the extraction algorithm’s dictionary. Specifically, the exposure of the study was given as “PFC”, and the exposure of interest (“PFOA” or “PFOS”) was only mentioned in the outcomes. The population was described as “maternal cord blood” rather than the population of interest (“pregnant women”). Therefore, all screening rules including *Any 2 PECO Terms* and EO failed to identify it as potentially relevant. This points at a limitation of the extraction algorithm that is also inherent in abstract and title screening in general and is not specific to screening by study characteristics.

### Limitations and future work

This study used three systematic reviews, and our reported performance may not generalise to other systematic reviews, especially in other domains, because of the small sample size. As more data becomes available, for example from efforts of the International Collaboration for the Automation of Systematic Reviews (ICASR), we will repeat this study to provide more robust conclusions.

Our study demonstrates the susceptibility of all automated screening methods to type I errors, i.e. erroneously excluding articles, as such decisions would not be revisited. Whilst some systems avoid type I errors by ranking articles rather than excluding them from further analysis, works saved by such systems are also limited. Further research is required on whether having a confidence measure, rather than a binary classification, would avoid such errors or whether another method could be more effective.

We have not investigated the reasons and implications of the EO screening rule providing best ones and hence did not make recommendations for more immediate changes to the systematic review process that could be made immediately. It may be possible that search strategies could be designed to require exposure and outcome terms to be included in the title and abstract and thus reduce the result set sizes without missing important studies. Further research comparing such search strategies, is still required.

## Conclusion

We have demonstrated a novel method for accelerating the screening for systematic reviews. Study characteristic extraction is done ahead of the screening, whilst expensive without automation is nonetheless practical and effective when such a method is automated.

## Appendix 1

### Extraction algorithm

The extraction algorithm used in this study uses rules and dictionaries developed using GATE (http://gate.ac.uk). GATE is a text mining desktop application that provides a graphical user interface and common text mining functions such as word and sentence tokenization.

Seventeen dictionaries were developed for the dataset. The dictionaries contain synonyms for the purpose of identifying study characteristics. The dictionaries used in by the algorithm are summarised in Table 5.

**Table 5:** Summary of dictionaries used in the extraction algorithm

Dictionary name	Size	Description	Example
Adjectives	32	Descriptive adjectives for the participant population	Non-smoking, postmenopausal
Controls	70	Nouns that refer to the participant population	Participants, students
Countries	464	Names of countries worldwide along with their respective nationalities	Malaysian, USA, American
Effect	41	Utilised epidemiological study metrics	Hazard ratio, adjusted odds ratio
Numbers	178	Numbers described by words	One, thousand
Related	24	Verbs indicating an association between exposure and outcome	Related, linked
Relations	26	Nouns indicating an association between exposure and outcome	Correlation, association
States	56	Names of the states and territories in the USA	Missouri, New York
Study types	21	Various epidemiological study designs (both observational and experimental)	Placebo-controlled, case control
PFOA	51	Various mentions of perfluorooctanoate (PFOA) mentions	PFOA, perfluorooctanoic acid
BPA	56	Variations of bisphenol A (BPA) mentions	BPA, urinary BPA concentrations
Folic acid	57	Variations of folic acid mentions	Folic acid, folic acid supplement
Air pollutants	73	Variations of outdoor particulate matter exposure mentions	Ambient no2, particulate air pollution
Fetal growth	87	Variations of fetal growth mentions	Birth weight, ponderal index
Twinning	28	Variations of twinning mentions	Twinning, twin pregnancies
Lung cancer	229	Variations of lung cancer and related comorbidities mentions	lc, lung cancer
Confounders	74	Various concept mentions as confounders in	Mode of delivery, fertility treatment use

The dictionaries were used in semantic rules that were used to identify specific phrases that correspond to a study characteristics. Each ruler maps to one characteristic but multiple rules can map to the same characteristics. Selection rules for each characteristic are given in Table 6.

**Table 6:** Selection of semantic rules used for extraction in GATE format, each with example phrases they match

Characteristic							
Study design	Example	We	conducted	a	hospital-based	prospective cohort	study
	Rule		(verb)	({Token.string==~“(?i)a”}|{Token.string==~“(?i)an”})	({Token})[0,1]	(study)	(types)?
Population	Example	Cohort			of	665 Danish pregnant women	
	Rule	({Token.string==~“(?i)cohort”}|{Token.string==~“(?i)sample”}|{Token.string==~“(?i)total”}|{Token.string==~“(?i)samples”}|{Token.string==~“(?i)cross-sectional”}|{Token.string==~“(?i)subsample”})			{Token.string==~“(?i)of”}	(population)	
Exposure	Example	Perfluorinated compounds		in relation to	birth weight		
	Rule	(folic_variations)		{Lookup.majorType==“relations”}	(birthoutcomes)		
Outcome	Example	association		with		miscarriage	
	Rule	{Lookup.majorType==“relations”}		({Token.string==~“(?i)between”}|(with))	({Token.string==~“(?i)the”})?	(birthoutcomes)	
Confounding factor	Example	Adjusting	for			covariates, including maternal pre-pregnancy BMI, smoking, education, and birth weight	
	Rule	(adjustment)	({Token.string = ~“(?i)for”}|{Token.string = ~“(?i)by”})		({Token})[0,3]	(cnf)	
Country	Example	In	Alberta	,	Canada		
	Rule	{Token.string==~“(?i)in”}	({Token}) [1]	{Token.string==“,”}	({Lookup.majorType==“countries”}|{Lookup.majorType==“states”})		

## Appendix 2

### Search strategies

Hamra 2014.

We repeated the search using the method section of the review.

Search: The following PubMed query was devised:

(((air pollution[Title/Abstract] OR particulate matter[Title/Abstract] OR traffic[Title/Abstract] AND cancer[Title/Abstract]))) AND (“1970/01/01”[Date - Entrez]: “2013/12/31”[Date - Entrez])

Citation tracking: Two hundred one additional papers were reported in the original review but were not available from the authors. The corresponding author of the review confirmed that all 201 articles were screened in the preliminary screening. We did not attempt to repeat citation tracking.

Fidelity: The original review does not report exactly how many articles were retrieved from the search before reports (we retrieve 683) but does report that 604 papers were left after initial filtering which is not specified exactly. We thus filtered the results using the following filter (“traffic” BUT NOT “air pollution”[ti]) which left 615 papers.

Johnson 2014.

The authors of this study have sent us the exported list of all 3023 articles screened, collected from 26 databases.

Thayer 2013.

Search: The original search queries were given to us by the corresponding author. The search date is not specified, nor how many papers remained after de-duplication.

Enmbase (excluding records from MEDLINE): “(“4,4 isopropylidenediphenol”/exp. OR “4,4 isopropylidenediphenol”:ti:ab OR “bisphenol A”/exp. OR “bisphenol A”:ti:ab OR 80–05-7:rn) AND (‘body weight disorder’/exp. OR obes*:ti:ab OR “body mass”:ti:ab OR ‘body weight’/exp. OR “body weight”:ti:ab OR “weight gain”:ti:ab OR overweight:ti:ab OR “body fat”:ti:ab OR adipocyte/exp. OR adipocyte*:ti:ab OR ‘lipid metabolism’/exp. OR lipid*:ti:ab OR adipogen*:ti:ab OR ‘adipose tissue’/exp. OR ‘adipocytokine’/exp. OR adipocytokine*:ti:ab OR adipokine*:ti:ab OR ‘adiponectin’/exp. OR adiponectin*:ti:ab OR adipos*:ti:ab OR ghrelin/exp. OR ghrelin:ti:ab OR leptin/exp. OR leptin:ti:ab OR resistin/exp. OR resistin:ti:ab OR lipogen*:ti:ab OR lipoprotein/exp. OR lipoprotein*:ti:ab OR triacylglycerol/exp. OR triacylglycerol:ti:ab OR triglyceride*:ti:ab OR ‘retinoid x receptor’/exp. OR RXR:ti:ab OR “retinoid x”:ti:ab OR “9-cis-retinoic”:ti:ab OR “peroxisome proliferator-activated receptors”/exp. OR PPAR*:ti:ab OR “peroxisome proliferator”:ti:ab OR glucocorticoid/exp. OR glucocorticoid*:ti:ab OR ‘liver x receptor’/exp. OR LXR:ti:ab OR “liver x”:ti:ab OR Nr1h2:ti:ab)” found 809 results.

PubMed: “(“4,4’ isopropylidenediphenol” OR “Bisphenol A” OR 80–05-7) AND (Obesity[mh] OR obes*[tiab] OR “body mass index”[mh] OR “body mass”[tiab] OR “body weight”[mh] OR “body weight”[tiab] OR “weight gain”[mh] OR “weight gain”[tiab] OR overweight[tiab] OR “body fat”[tiab] OR adipocyte[mh] OR adipocyte*[tiab] OR adipogenesis[mh] OR adipogen*[tiab] OR “adipose tissue”[mh] OR adipos*[tiab] OR adipokines[mh] OR adipokine*[tiab] OR adipocytokine*[tiab] OR adiponectin[mh] OR adiponectin*[tiab] OR ghrelin[mh] OR ghrelin[tiab] OR leptin[mh] OR leptin*[tiab] OR resistin[mh] OR resistin[tiab] OR Lipid metabolism[mh] OR lipogen*[tiab] OR lipid[tiab] OR lipids[tiab] OR lipoprotein OR triacylglycerol OR triglyceride OR “Retinoid x receptors”[mh] OR RXR[tiab] OR “retinoid x”[tiab] OR “9-cis-retinoic”[tiab] OR “peroxisome proliferator-activated receptors”[mh] OR PPAR*[tiab] OR “peroxisome proliferator”[tiab] OR “receptors, glucocorticoid”[mh] OR glucocorticoid*[tiab] OR “liver x receptor”[supplementary concept] OR LXR*[tiab] OR “liver x”[tiab] OR Nr1h2[tiab])” found 480 articles.

Scopus advanced search “TITLE-ABS-KEY(“4,4’ isopropylidenediphenol” OR “Bisphenol A” OR 80-05-7) AND TITLE-ABS-KEY(obes* OR “body mass” OR “body weight” OR “weight gain” OR overweight OR “body fat” OR adipocyte* OR adipogen* OR adipos* OR adipokine* OR adipocytokine* OR adiponectin* OR ghrelin OR leptin* OR resistin OR lipogen* OR lipid* OR lipoprotein* OR triglyceride* OR triacylglycerol OR RXR OR “retinoid x” OR “9-cis-retinoic” OR PPAR* OR “peroxisome proliferator” OR glucocorticoid* OR LXR* OR “liver x” OR Nr1h2)” found 734 articles.

Web of Science “TS=(“4,4’ isopropylidenediphenol” OR “Bisphenol A” OR 80-05-7) AND TS=(obes* OR “body mass” OR “body weight” OR “weight gain” OR overweight OR “body fat” OR adipocyte* OR adipogen* OR adipos* OR adipokine* OR adipocytokine* OR adiponectin* OR ghrelin OR leptin* OR resistin OR lipogen* OR lipid OR lipids OR lipoprotein* OR triglyceride* OR triacylglycerol OR RXR OR “retinoid x” OR “9-cis-retinoic” OR PPAR* OR “peroxisome proliferator” OR glucocorticoid* OR LXR* OR “liver x” OR Nr1h2)” found 633 articles.

Toxline excluding PubMed records and adding synonyms and CAS numbers “(“4,4’ isopropylidenediphenol” OR “Bisphenol A” OR 80-05-7) AND (obes* OR “body mass” OR “body weight” OR “weight gain” OR overweight OR “body fat” OR adipocyte* OR adipogen* OR adipos* OR adipokine* OR adipocytokine* OR adiponectin* OR ghrelin OR leptin* OR resistin OR lipogen* OR lipid OR lipids OR lipoprotein* OR triglyceride* OR triacylglycerol OR RXR OR “retinoid x” OR “9-cis-retinoic” OR PPAR* OR “peroxisome proliferator” OR glucocorticoid* OR LXR* OR “liver x” OR Nr1h2)” found 115 articles.

The reviewers also searched the EPA’s ACTOR database (https://actor.epa.gov/actor/home.xhtml) and Chemical Data Access Tool (http://java.epa.gov/oppt_chemical_search), PubChem and the Cochrane database which found no results.

Fidelity: Repeating these searches, we retrieved from Embase 290 articles, Scopus 978 articles, Toxline 351 articles and PubMed 435 articles. After de-duplication, 2054 articles remained.

## Abbreviations

C: Control (group)
E: Exposure
FN: False negatives
FP: False positives
FPR: False positive rate
GATE: General Architecture for Text Engineering
I: Intervention
O: Outcome or observation
P: Population
PFC: Perfluorinated chemicals
PFOA: Perfluorooctanoic acid
PFOS: Perfluorooctane sulfonate
Pr: Precision
Re: Recall
TN: True negatives
TP: True positives

## References

[1] Tsafnat G, Dunn A, Glasziou P, Coiera E (2013). The automation of systematic reviews. BMJ.

[2] Elliott JH, Turner T, Clavisi O, Thomas J, Higgins JP, Mavergames C, Gruen RL (2014). Living systematic reviews: an emerging opportunity to narrow the evidence-practice gap. PLoS Med.

[3] Tsafnat G, Glasziou P, Choong MK, Dunn A, Galgani F, Coiera E (2014). Systematic review automation technologies. Systematic reviews.

[4] Wilczynski NL, McKibbon KA, Haynes RB (2011). Sensitive clinical queries retrieved relevant systematic reviews as well as primary studies: an analytic survey. J Clin Epidemiol.

[5] O’Mara-Eves A, Thomas J, McNaught J, Miwa M, Ananiadou S (2015). Using text mining for study identification in systematic reviews: a systematic review of current approaches. Systematic reviews.

[6] Ouzzani M, Hammady H, Fedorowicz Z, Elmagarmid A (2016). Rayyan—a web and mobile app for systematic reviews. Systematic reviews.

[7] Tsafnat G, Glasziou P, Dunn A, Coiera E (2014). Systematic-review automation technologies. Systematic reviews.

[8] Mishra R, Bian J, Fiszman M, Weir CR, Jonnalagadda S, Mostafa J, Del Fiol G (2014). Text summarization in the biomedical domain: a systematic review of recent research. J Biomed Inform.

[9] Rada G, Pérez D, Capurro D (2013). Epistemonikos: a free, relational, collaborative, multilingual database of health evidence. MedInfo.

[10] Karystianis G, Thayer K, Wolfe M, Tsafnat G (2017). Evaluation of a rule-based method for epidemiological document classification towards the automation of systematic reviews. J Biomed Inform.

[11] Ananiadou S, Kell DB, Tsujii J-I (2006). Text mining and its potential applications in systems biology. Trends Biotechnol.

[12] Olorisade BK, de Quincey E, Brereton P, Andras P: A critical analysis of studies that address the use of text mining for citation screening in systematic reviews. In: Proceedings of the 20th International Conference on Evaluation and Assessment in Software Engineering: 2016. Limerick: ACM; 2016. p. 14.

[13] Fromme H, Mosch C, Morovitz M, Alba-Alejandre I, Boehmer S, Kiranoglu M, Faber F, Hannibal I, Genzel-Boroviczény O, Koletzko B (2010). Pre-and postnatal exposure to perfluorinated compounds (PFCs). Environmental science & technology.

[14] Hamra GB, Guha N, Cohen A, Laden F, Raaschou-Nielsen O, Samet JM, Vineis P, Forastiere F, Saldiva P, Yorifuji T (2014). Outdoor particulate matter exposure and lung cancer: a systematic review and meta-analysis. Environ Health Perspect.

[15] Johnson PI, Sutton P, Atchley DS, Koustas E, Lam J, Sen S, Robinson KA, Axelrad DA, Woodruff TJ (2014). The navigation guide—evidence-based medicine meets environmental health: systematic review of human evidence for PFOA effects on fetal growth. Environ Health Perspect.

[16] Thayer K, Rooney A, Boyles A, Holmgren S, Walker V, Kissling G. Draft protocol for systematic review to evaluate the evidence for an association between bisphenol A (BPA) exposure and obesity. National Toxicology Program. 2013.

